# 20-kHz alternating current stimulation: effects on motor and somatosensory thresholds

**DOI:** 10.1186/s12984-020-00661-x

**Published:** 2020-02-19

**Authors:** Diego Serrano-Muñoz, Juan Avendaño-Coy, Cristina Simón-Martínez, Julian Taylor, Julio Gómez-Soriano

**Affiliations:** 1 Sensorimotor Function Group, Hospital Nacional de Parapléjicos, 45071 Toledo, Spain; 2 Toledo Physiotherapy Research Group (GIFTO), Faculty of Physiotherapy and Nursery, Castilla La Mancha University, 45071 Toledo, Spain; 3 Department of Rehabilitation Sciences, KU Leuven - University of Leuven, 3000 Leuven, Belgium

**Keywords:** Nerve block, High-frequency, Transcutaneous electric stimulation, Peripheral nerve

## Abstract

**Background:**

High frequency alternating current (HFAC) stimulation have been shown to produce a peripheral nerve conduction block. Currently, all the studies applying HFAC stimulation in clinical studies, have employed frequencies below 10 kHz. The main aim of this work was to investigate the neuromodulatory effect of transcutaneous 20 kHz stimulation on somatosensory and pain thresholds, and maximal handgrip strength.

**Methods:**

A randomized, crossover, single-blinded, placebo-controlled trial was conducted following recruitment of fourteen healthy volunteers. Transcutaneous stimulation at 20 kHz and sham stimulation were applied over the ulnar and median nerves of fourteen healthy volunteers for 20 min. Maximal handgrip strength (MHS), mechanical detection threshold (MDT) and pressure pain threshold (PPT) were registered prior to, during (15 min), immediately after the end (20 min), and 10 min following stimulation.

**Results:**

The 20 kHz stimulation showed a lower MHS during the stimulation at the 15 min (30.1 kgs SE 2.8) and at 20 min (31.8 kgs, SE 2.8) when compared to sham stimulation (35.1 kgs, SE 3.4; *p* < 0.001 and 34.2 kgs, SE 3.4; *p* = 0.03, respectively). The 20 kHz stimulation resulted in a slight increase in MDT at 15 min (0.25 mN; 0.25–2.00) when compared to the sham stimulation (0.25 mN; 0.25–0.25; *p* = 0.02), and no effects were showed for PPT.

**Conclusions:**

High-frequency stimulation at 20 kHz suggests a partial block of nerve activity. Studies in subjects with neurological disorders characterized by nerve hyperactivity are needed to confirm the clinical impact of this non-invasive electrical stimulation technique.

**Trial registration:**

NCT, NCT02837458. Registered on 12 April 2017.

## Background

Many neurological pathologies lead to neuromuscular dysfunction characterized by an abnormal increase in neural activity, such as the spasticity syndrome or chronic evoked pain [1]. Exaggerated neuromuscular activity requires therapeutic treatment, which may be based on non-pharmaceutical strategies, including neuromodulation using electrical stimulation to reduce neuronal hyperexcitability. High-frequency alternating current (HFAC) stimulation applied at frequencies higher than 1 kHz have been shown to produce a peripheral nerve conduction block in preclinical studies, an effect which is rapidly reversed after stimulation [2]. Studies using experimental models have demonstrated that the minimum stimulation frequency required to produce a conduction block without causing damage to the peripheral nerve is approximately within the range of 4–5 kHz [3–5]. Although HFAC has been shown to be a safe technique, only a few studies using this stimulation protocol has been applied to humans [6–11]. The application of HFAC stimulation using implanted devices reveal positive therapeutic effects with regards to reducing body weight following the intermittent conduction block of vagal nerve activity in obese people [8, 12], in reducing tinnitus after auditory nerve block with a HFAC applied at 5 kHz [13], and also in reducing pain using 10 kHz following limb amputation [9].

To date only four published studies have reported the effects of non-invasive transcutaneous HFAC stimulation over peripheral nerves [7, 10, 11, 14]. In a previous study conducted by our group, 5 kHz HFAC stimulation applied over the radial nerve for 20 min produced similar modulatory effects on somatosensory thresholds when compared to transcutaneous electrical nerve stimulation (TENS) [7]. Another pilot study demonstrated a larger decrease in maximal handgrip strength during 10 kHz HFAC of the median and ulnar nerve, when compared to either a 5 kHz or sham stimulation [14]. Similar results have been shown by Kim et al. [10], reported an increase in mechanical detection threshold (MDT) and pressure pain threshold (PPT) after 10 kHz HFAC stimulation of the median nerve, and an additional decrease in finger muscle contraction during the stimulation. Finally, Springer et al. [11], who showed that the application of 7 kHz HFAC to the ulnar nerve resulted in a reduction in muscle activity of the abductor digiti minimi, as measured with electromyography, immediately after HFAC stimulation.

Altogether, these clinical experimental studies support the potential effect of HFAC stimulation on nerve conduction block, even though the optimal stimulation frequency remains unknown. Currently, all the studies applying HFAC stimulation in clinical studies [15], using either invasive electrodes or transcutaneous techniques, have employed frequencies below 10 kHz. However, a study performed by Ackermann et al. [16] in non-human primates, whose nerve diameter is similar to humans, showed that the most effective stimulation frequency was in the range of 20–40 kHz, with the 10 kHz stimulation protocol being ineffective for nerve conduction block. More animal studies have observed that frequencies higher than 10 kHz was more effective for obtaining partial nerve block [17–19].

To date no clinical study has employed transcutaneous HFAC at frequencies greater than 10 kHz. Therefore, the main objective of this study was to investigate the neuromodulatory effect of transcutaneous HFAC stimulation applied on the median and ulnar nerve at a frequency of 20 kHz on somatosensory and pain thresholds and maximal handgrip strength in healthy participants, compared to sham stimulation.

## Methods

### Design

A randomized, crossover, single-blinded, placebo-controlled trial was conducted following recruitment of fourteen healthy volunteers. All volunteers signed the informed consent form approved by the Local Ethics Committee (Ref. No. 158; 02/11/2017). The present study was registered in the ClinicalTrials.gov Protocol Registration System (NCT02837458). Participants received both interventions (20 kHz and sham stimulation), and the order of these interventions was randomized using a web page tool (www.randomizer.org). The duration of the active or sham intervention was 20 min. The evaluation included measures A, B and C (see section *D. Outcome measures*) assessed throughout the experiment at four time points: i) before stimulation (0 min), ii) during stimulation at 15 min (15 min), iii) at the end of the stimulation (20 min) and iv), at 10 min after the end of the stimulation (30 min). A washout period of 24 h was used between the application of the two interventions [14].

### Subjects

Healthy volunteers between 18 and 65 years old, without any peripheral or central nervous system pathology, were recruited using non-probabilistic convenience sampling. The exclusion criteria included musculoskeletal pathology in the upper limb, inability to tolerate electrical current stimulation, allergy to the stimulating electrode material, pacemaker or any other implanted device, epilepsy, neurotrauma, recent surgical procedures, diabetes, pregnancy, and cancer.

### Intervention

Subjects were seated with their dominant elbow flexed at 90°. Two surface self-adhesive electrodes 5 cm × 5 cm (ValuTrode, Axelgaard Manufacturing Co, LTD, Fallbrook, USA) were used. The proximal electrode was fixed to the skin over the path of the ulnar nerve at the epitrochlea and the distal electrode was placed on the median nerve over the carpal tunnel [14]. The same stimulator device was used to apply the two interventions (Myomed 932, Enraf-Nonius, Delft, Netherlands), whose software was previously modified to be able to deliver an electrical current of 20 kHz. Interferential tetrapolar mode was used, although only two electrodes (Channel 1) was connected. The other two electrodes (Channel 2) were not used to avoid any affect due to interference. A digital oscilloscope (Tektronix TDS2014B, Beaverton, USA) was used to confirm that the pulse was biphasic sinusoidal waveform with no modulation and with a frequency of 20 kHz.

#### 20 kHz stimulation

A charge-balanced, symmetric, biphasic sinusoidal current without modulation was presented at a frequency of 20 kHz for the active intervention. The stimulation intensity was defined as that sufficient to produce a “strong but comfortable” sensation, just below motor threshold [20]. The intensity was gradually increased until a minimal visible contraction was observed and then subsequently decreased until the muscle contraction disappeared, with the same intensity maintained throughout the stimulation. To avoid habituation to the stimulus, participants were asked to corroborate the perceived stimulation sensation every 2 min, so that the stimulation intensity could be increased if requested [21, 22].

#### Sham stimulation

Sham stimulation was applied with the same device and electrode placement, but the intensity was adjusted by simulating to progressively increase the intensity of a non-connected channel. Participants were also blinded to the hypothesis of the study by being informed that in some cases the perceived sensation might be of different as the stimulus intensity could be adjusted to below their sensory threshold, with the possibility that the participant may or may not feel the stimulus current [7, 20].

### Outcome measures

#### Maximal handgrip strength (MHS)

**Fig. 1:**
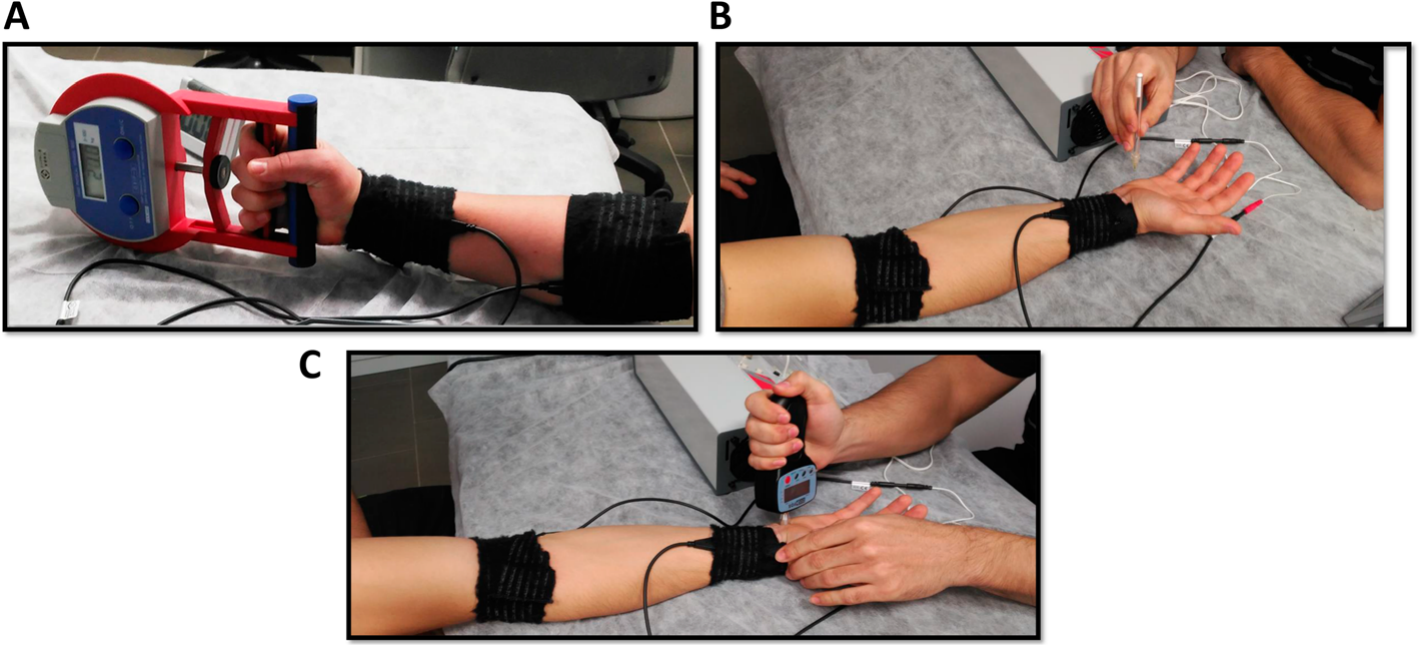
Stimulus intervention, electrode placement and outcome measure assessment. **a** Maximal handgrip strength. **b** Mechanical detection threshold. **c** Pressure pain threshold

Maximal voluntary handgrip muscle strength was measured using a handgrip dynamometer (Grip Strength Dynamometer T.K.K. 5401 GRIP-D Takei Scientific Instruments CO., LTD. Shinagawa-ku, Tokyo). Average maximal handgrip muscle strength was calculated as mean of three measurements and used for statistical analysis [14] (Fig. 1a). The MHS outcome was reported in kilograms.

#### Mechanical detection threshold (MDT)

Mechanical sensory threshold was examined at the palmar surface of the hand, over a 1 cm^2^ surface area at the hypothenar eminence. The MDT was assessed using modified Von Frey filaments (OptiHair2, MARSTOCKnervtest, Marburg, Germany), using the following calibrated filaments to apply forces of 0.25, 0.5, 2, 4, 8, 16, 32, 128, 256 y 512 mN [23]. Participants were instructed to close their eyes during the sensory examination and say ‘yes’ when perceived the sensory stimulus. The sensory examination involved seven applications for each filament. MDT was defined when sensory perception was successful for at least 4 of the 7 times (Fig. 1b).

#### Pressure pain threshold (PPT)

The PPT was assessed with the application of a digital algometer (Wagner Instruments, FORCE ONE model FDIX, USA) applied on the triquetral bone. The stimulus was presented as a circular stimulus tip of 1 cm in diameter, measured on a digital interval scale of 0.1 N. Mechanical pressure was applied at a rate of 5 N/s [24] and the mean PPT was calculated as the average of three consecutive measurements (Fig. 1c). The outcome is reported in Newtons.

### Statistical analysis

Descriptive statistics are reported in mean and standard error or median and interquartile range according to the data distribution (normal or non-normal distribution, respectively). Statistical analysis was performed with a commercial software package Sigmaplot 12.0 for Windows (Systat software, Inc., Germany). The estimated sample size for the study was calculated using MHS as the main variable [14]. Taking into consideration a mean difference between sham and active intervention groups of 15%, a standard deviation of 15%, with a type I error (α) of 0.05 and a power of 80%, a sample size of 10 subjects was calculated. However, as a contingency, the sample size was increased by four more subjects (*n* = 14). The Gaussian distribution of the data was confirmed with the Shapiro-Walk test, and parametric statistical tests were adopted. A two-way repeated-measures ANOVA, with the “time” factor (0 min, 15 min, 20 min, 30 min) and the “intervention” factor (20 kHz and sham stimulation), was performed to compare differences in MHS and PPT. Post-hoc analysis test was assessed with the Bonferroni test. Because MDT is a non-parametric measure, Friedman test was performed to analyse differences within time points (0 min, 15 min, 20 min, 30 min). U-Mann Whitney test was used to compare both interventions (20 kHz and sham stimulation). Post-hoc analysis test was assessed with the Tukey test. A *p* value of < 0.05 was considered statistically significant.

## Results

Fourteen healthy volunteers completed the study 7 (50%) males, mean age 33.3 years (SD 6.8) ranged between 23 and 44 years. No adverse effects were reported by any participant. The mean current intensity applied at the onset of the stimulus was 44.2 mA (SD 12.8), and the mean final intensity was 85.0 mA (SD 11.4; *p* < 0.001).

### Maximal handgrip strength (MHS)

**Fig. 2:**
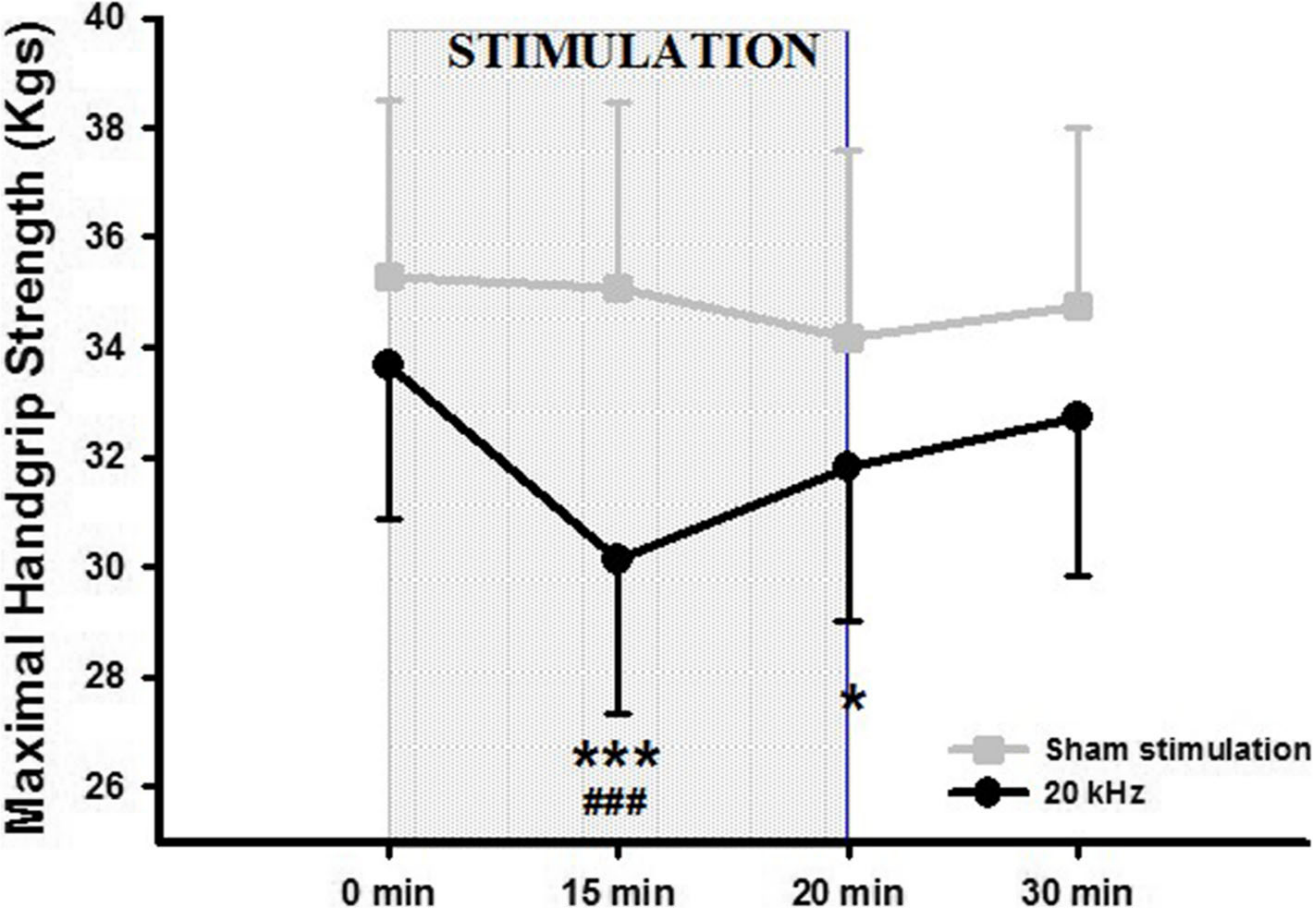
HFAC stimulation effect on maximal handgrip strength. Sham stimulation (grey) and 20 kHz (black). Data are represented as mean and standard error values. * Indicates value significantly different compared to sham stimulation (***p < 0.001; * *p* < 0.05). # Indicates significant difference from baseline and 30 min (### *p* < 0.001)

Significant differences in the “time” factor (F_(3,39)_ = 4.09; *p* = 0.013), the “intervention” factor (F_(1,13)_ = 12.14; *p* = 0.004) and the “time-intervention” interaction (F_(3,39)_ = 4.09; p = 0.013) were detected. Specifically, 20 kHz achieved a decrease in MHS at 15 min during the intervention (30.1 kgs, SE 2.8) when compared with baseline (33.7 kgs, SE 2.8; *p* < 0.001) and to the 10 min post-stimulation time point (32.7 kgs, SE 2.9; *p* < 0.01). Sham stimulation did not show a significant change compared to baseline. A comparison of the both interventions revealed that the 20 kHz stimulus produced a lower MHS (30.1 kgs, SE 2.8) when compared to sham stimulation (35.1 kgs, SE 3.4; *p* < 0.001) during the stimulation at the 15 min test period. Immediately after the intervention at 20 min with 20 kHz stimulation, MHS was statistically lower (31.8 kgs, SE 2.8) when compared to the same measure with sham stimulation (34.2 kgs, SE 3.4; *p* = 0.03). (Fig. 2).

### Mechanical detection threshold (MDT)

A statistically significant difference was found in the comparison of median values of 20 kHz intervention among time points (*p* < 0.01), however the post-hoc test used did not reveal any significant difference. No statistical change in MDT was detected within sham stimulation. When the effect of active or sham stimulation were compared, the 20 kHz stimulus revealed a slight increase in MDT (0.25 mN; 0.25–2.00) at 15 min during the stimulation when compared to the sham stimulation (0.25 mN, 0.25–0.25; *p* = 0.02) (Table 1).

**Table 1:** Changes in mechanical detection (mN) and pressure pain threshold (N/cm2)

	Intervention	0 min	15 min	20 min	30 min	Significance intragroup ^(a)^
Mechanical Detection Threshold	20 kHz	0.25 (0.25–0.25)	0.25 (0.25–2.00)	0.25 (0.25–0.25)	0.25 (0.25–0.25)	χ^2^: 12,75; *p* < 0.01
	SHAM	0.25 (0.25–0.25)	0.25 (0.25–0.25)	0.25 (0.25–0.25)	0.25 (0.25–0.25)	χ^2^: 0,00; *p* = 1.00
	Significance intergroup ^(b)^	*p* = 1.00	*p* = 0.02	*p* = 0.36	*p* = 1.00	
Pressure Pain Threshold ^(c)^	20 kHz	70.0 (4.3)	69.8 (4.2)	70.5 (4.9)	68.9 (4.8)	“Time” factor: F: 0.54; *p* = 0.66 “Intervention” factor: F: 0.26; *p* = 0.62
	SHAM	69.0 (4.6)	70.0 (5.2)	67.3 (4.9)	68.5 (5.2)	

Data of Mechanical Detection Threshold are expressed as median values (25–75% inter-quartile range), and data of Pressure Pain Threshold as mean value (standard error). ^(a)^Friedman test. ^(b)^ Mann-Whitney test. ^(c)^ Two-way ANOVA repeated measures

### Pressure pain threshold (PPT)

No significant differences were found in any comparison for PPT, taking into consideration the “time” factor (F_(3,39)_ = 0.54; *p* = 0.66), the “intervention” factor (F_(1,13)_ = 0.26; *p* = 0.62), or the “intervention-time” intersection (F_(3,39)_ = 0.84; *p* = 0.48) (Table 1).

## Discussion

This is the first study showing the application of 20 kHz stimulation in healthy participants over somatosensory and motor outcome measures. The main results of the present study showed that the application of transcutaneous HFAC at 20 kHz over the median and ulnar nerves in healthy participants decreased maximal handgrip strength and slightly increased mechanical detection threshold when compared to sham stimulation. These results suggest that 20 kHz stimulation could evoke a partial nerve conduction block, which has been previously demonstrated using a 10 kHz stimulus [10, 14]. However, although the increase in MDT was statistically significant, the change was not determined to be clinically significant (Table 1). In addition, the pressure pain threshold did not change after the application of the stimulation. The difference of effect of 20 kHz on specific sensory modalities may be due to specific effects on nerve fiber types as each has a different conduction block threshold, which in turn varies with the block frequency, observed in preclinical studies [25].

Previous studies have shown inhibited motor activity, Kim et al. [10] applied 10 kHz HFAC stimulation over the median nerve and observed a decrease in finger muscle contraction of 40%. Springer et al. [11] applied 7 kHz stimulation over the ulnar nerve and observed a decrease of 8% in maximal voluntary contraction and a 15% decrease in the myoelectrical activity of the abductor digiti minimi muscle, immediately after HFAC stimulation, indicating an effect on motor activity. The decrease in maximal handgrip strength with 20 kHz HFAC stimulation observed in the present study (11%) is in line with our previous study at 10 kHz [14], where the reduction in MHS was of 14%, at the same test times. The stimulation of proprioceptive fibers with the electrical stimulation may interfere with the accurate perception and performance of maximal voluntary muscle contraction under the test conditions, and may constitute one of the reasons why a reduction in muscle strength observed in this study.

Moreover, an increase in tactile and pressure pain threshold was also detected in previous studies at 5 and 10 kHz, indicating decreased sensory function. Our previous study when HFAC stimulation was presented at 5 kHz to the radial nerve [7] the increase of MHS was of 17.2 mN. Kim et al. [10] with 10 kHz observed an increase MDT to approximately twice that of the baseline condition during the stimulation. However, in our study with 20 kHz, HFAC stimulation was only able to block selectively motor activity assessed as MHS, with a slight increase on the threshold of Aβ fibers assessed by MDT, and without producing any significant differences on Aδ sensory fibers, assessed as PPT. Because of this slight change on MDT, where the median value is the same, it could be supposed that the clinical effect could be not significant. This selective effect on motor activity could have interesting clinical implications, for example in people with the spasticity syndrome as the stimulation protocol would be able to specifically modulate the motor component without producing large changes in sensory pathways. This study also suggests that transcutaneous application of HFAC at 20 kHz does not produce complete nerve conduction block in healthy volunteers, compared to the effect of direct nerve stimulation performed in preclinical studies using implanted electrodes [5, 26–28].

The discrepancy between the nerve conduction blockade in the animal and human studies could be explained by the greater distance between the stimulating electrode and the axon target during transcutaneous HFAC stimulation. However, the partial conduction block observed in our study, is in line with previous transcutaneous stimulation studies in human subjects [7, 10, 11, 14]. Of clinical relevance, our study showed that the effects of the stimulation were reverted immediately after the end of the stimulation, which is in consonance with previous preclinical studies [15]. In an “in vivo” study, the total time for recovery of normal function after stimulation lasted between 300 ms to 60 s [29]. Furthermore, an “in vitro” study reported that recovery of nerve function ranged between 4 to 10 min, and also found that small diameter fibers recovered faster (C-fiber: 3 min; Aδ fiber: 6 min) [30]. These results suggest that stimulation protocols designed to block nerve conduction may have a preferential effect on motor fibers whose diameter are larger than smaller Aδ and C fibers, in addition to longer recovery time following nerve conduction block.

In this study, none of the participants reported adverse effects when HFAC was applied, and the modulation of motor and sensory nerve activity was quickly reversible after the stimulus, suggesting that the transcutaneous HFAC stimulation protocol was safe. Following several neuropathologies an aberrant increase in nerve activity may lead to specific symptoms of spasticity such as hypertonia, spasms, tremors, chronic pain could be reduced by the transcutaneous application of 20 kHz HFAC by mediating a nerve conduction block, although the optimal stimulation frequency remains unknown. HFAC stimulation could have a great potential as a therapeutic tool for pathologies characterized by a hyperactivity of the nervous system, with the aim of inhibiting exaggerated nervous activity.

### Study limitations

The main limitation of this study is that the sensory measures used are considered as quasi-objective, as their quantification relies on the perceived sensation and judgement of the participant. Neurophysiological measures such as motor and somatosensory evoked potentials may have more potential to objectively evaluate changes in nerve conduction during and after the stimulation. Another limitation is that the assessor was not blinded to the application of each intervention (20 kHz or sham stimulation). With regards to blinding procedures for subjects, the specific method used in this study has been used before [7, 20], although the success of the blinding technique was not formally tested. Finally, the present study was performed in healthy subjects and therefore conclusions about the applicability of the HFAC stimulation technique in patients should be made with caution until further studies are developed in participants with neurological disorders characterized by motor hyperactivity.

## Conclusions

High frequency alternating current stimulation at a frequency of 20 kHz applied over the ulnar and median nerves of healthy volunteers produces a decrease in muscle handgrip strength during the stimulation and a slight change in mechanical detection threshold, when compared to sham stimulation. HFAC at 20 kHz has potential for clinical applications where a selective stimulation protocol is required to partially block alpha-motoneurons without producing large changes in the sensory pathways.

## Data Availability

The data collected in this study are available from the corresponding author on reasonable request.

## Abbreviations

ANOVA: Analysis of variance
HFAC: High-frequency alternating current
MDT: Mechanical detection threshold
MHS: Maximal handgrip strength
PPT: Pressure pain threshold
SD: Standard deviation
SE: Standard error
TENS: Transcutaneous electrical nerve stimulation
